# Diabetic mice have retinal and choroidal blood flow deficits and electroretinogram deficits with impaired responses to hypercapnia

**DOI:** 10.1371/journal.pone.0259505

**Published:** 2021-12-09

**Authors:** Eric R. Muir, Divya Narayanan, Saurav B. Chandra, Nikolay P. Akimov, Jeong-Hyeon Sohn, Evan Meyer, René C. Rentería, Timothy Q. Duong

**Affiliations:** 1 Department of Radiology, Stony Brook University, Stony Brook, NY, United States of America; 2 Department of Ophthalmology, University of Texas Health San Antonio, San Antonio, TX, United States of America; 3 Research Imaging Institute, University of Texas Health San Antonio, San Antonio, TX, United States of America; 4 Department of Biomedical Engineering, Stony Brook University, Stony Brook, NY, United States of America; 5 Center for Biomedical Neuroscience, University of Texas Health San Antonio, San Antonio, TX, United States of America; 6 Department of Clinical and Applied Science Education, School of Osteopathic Medicine, The University of the Incarnate Word, San Antonio, TX, United States of America; 7 Department of Radiology, Albert Einstein College of Medicine, Bronx, NY, United States of America; Transilvania University of Brasov: Universitatea Transilvania din Brasov, ROMANIA

## Abstract

**Purpose:**

The purpose of this study was to investigate neuronal and vascular functional deficits in the retina and their association in a diabetic mouse model. We measured electroretinography (ERG) responses and choroidal and retinal blood flow (ChBF, RBF) with magnetic resonance imaging (MRI) in healthy and diabetic mice under basal conditions and under hypercapnic challenge.

**Methods:**

Ins2^Akita^ diabetic (Diab, n = 8) and age-matched, wild-type C57BL/6J mice (Ctrl, n = 8) were studied under room air and moderate hypercapnia (5% CO_2_). Dark-adapted ERG a-wave, b-wave, and oscillatory potentials (OPs) were measured for a series of flashes. Regional ChBF and RBF under air and hypercapnia were measured using MRI in the same mice.

**Results:**

Under room air, Diab mice had compromised ERG b-wave and OPs (e.g., b-wave amplitude was 422.2±10.7 μV in Diab vs. 600.1±13.9 μV in Ctrl, p < 0.001). Under hypercapnia, OPs and b-wave amplitudes were significantly reduced in Diab (OPs by 30.3±3.0% in Diab vs. -3.0±3.6% in Ctrl, b-wave by 17.9±1.4% in Diab vs. 1.3±0.5% in Ctrl). Both ChBF and RBF had significant differences in regional blood flow, with Diab mice having substantially lower blood flow in the nasal region (ChBF was 5.4±1.0 ml/g/min in Diab vs. 8.6±1.0 ml/g/min in Ctrl, RBF was 0.91±0.10 ml/g/min in Diab vs. 1.52±0.24 ml/g/min in Ctrl). Under hypercapnia, ChBF increased in both Ctrl and Diab without significant group difference (31±7% in Diab vs. 17±7% in Ctrl, p > 0.05), but an increase in RBF was not detected for either group.

**Conclusions:**

Inner retinal neuronal function and both retinal and choroidal blood flow were impaired in Diab mice. Hypercapnia further compromised inner retinal neuronal function in diabetes, while the blood flow response was not affected, suggesting that the diabetic retina has difficulty adapting to metabolic challenges due to factors other than impaired blood flow regulation.

## Introduction

Diabetic retinopathy (DR) is considered primarily a microvascular disease of the retina and is clinically diagnosed and monitored based on visually apparent signs of vascular damage, such as microaneurysms and cotton wool spots. Chronic hyperglycemia in diabetes injures retinal microvascular endothelial cells, pericytes, and neurons [1,2]. The diabetic milieu (including, for example, dyslipidemia, inflammatory activation, decreased insulin signaling, and hyperglycemia) causes metabolic disruption to which the retina incompletely adapts, leading to dysfunction of multiple cell types and affecting their interactions [3].

Increasing evidence from human [4] and animal models of DR [5] indicates that injury to retinal neurons, in addition to microvascular dysfunction, plays a crucial role early in DR pathology, prior to observable clinical manifestations. For example, recent reports in the Ins2^Akita^ mouse, a model of type 1 diabetes that develops retinopathy [6], have shown that the b-wave and oscillatory potentials (OPs) of the electroretinogram (ERG) are impaired after several months of diabetes [7,8]. Further, we have shown that both visual function (the optokinetic tracking reflex) and ocular blood flow are impaired after several months of diabetes in Ins2^Akita^ mice, with the vascular deficit in the choroid detected earlier, consistent with the idea that vascular disruption alters function early in the disease [9]. However, the association between retinal and choroidal blood flow deficits and neuronal dysfunction in diabetes remains relatively unexplored.

In the normal retina, vascular metabolic regulation couples blood flow to neuronal function to provide sufficient support for the tissue’s metabolic demands, driven at least in part by CO_2_ produced as a metabolic by-product [2,10]. There are substantial differences in regulation of retinal and choroidal blood flow [11]. The retinal vasculature is regulated locally by the metabolic needs of the tissue it perfuses, while the choroid is not regulated by local metabolic needs, as it is separated from the outer retina, the tissue that it nourishes. Oxygen inhalation decreases retinal blood flow [12], while having no effect on choroidal blood flow [13,14]. Hypercapnia increases blood flow in both the retina and choroid [13,15], while hypocapnia decreases blood flow in both circulations [16]. Vascular regulation in the retina has been frequently studied using hypercapnic challenge to induce vasodilation and increase blood flow independent of neuronal stimulation [2,13,17,18]. Evidence indicates that vascular responses to metabolic conditions in diabetic eyes are perturbed [2,19], and human diabetic patients reportedly have reduced retinal vascular reactivity to CO_2_ inhalation [20]. While increased blood flow may potentially improve retinal function in vascular diseases, hypercapnia has other physiological effects on the retina, such as reducing pH, which may cause metabolic stress and compromise vision [21–23]. The ERG response can be reduced by hypercapnia in normal retinas [22,24,25], while hypercapnia reduces contrast sensitivity in glaucoma patients but not normal subjects [26]. However, the effects of hypercapnia on the function of the diabetic retina, in which basal metabolism is already altered and vascular reactivity may be impaired, are unknown.

The purpose of this study was to investigate neuronal and vascular functional deficits in the retina and their association in the Ins2^Akita^ mouse model of diabetes. We determined scotopic ERG responses and used MRI to measure retinal blood flow (RBF) and choroidal blood flow (ChBF) in normal and diabetic mice. Data were recorded under two conditions: when the animals were breathing normal room air and under hypercapnia (i.e., breathing air mixed to 5% CO_2_). Our hypothesis was that ERG deficits in diabetic mice would be correlated to ocular blood flow and that diabetic mice would have associated abnormal ERG and blood flow responses to hypercapnia compared to normal mice. However, we found that while diabetic mice had greater impairment of ERG parameters than control mice under hypercapnia, the blood flow responses to hypercapnia in diabetic mice were similar to those in control mice.

## Material and methods

### Animals

Experiments were conducted on 8 months old, heterozygous male Ins2^Akita^ diabetic mice (Diab, n = 8) and age-matched, normal, C57BL/6J mice (Ctrl, n = 8) (Jackson Laboratory, Bar Harbor, ME). ERG and MRI was performed on the same groups with ERG on all animals and MRI on 7 Ctrl and 8 Diab. However, MRI for one Diab animal was excluded as the eye appeared deflated on MRI with distorted retina, although the eye appeared normal under slit lamp examination at the time of ERG. Animals were housed in our institutional animal facilities in typical mouse cages under a 12/12 light/dark cycle and received a standard rodent diet with 5.7% fat and 20% protein (Teklad LM-485; Harlan, Houston, TX). Lighting was from overhead fluorescent bulbs typical of such facilities and reached approximately 20 Cd/m^2^ when measured with a photometric detector (UDT Instruments) facing the room from within a mouse cage in the middle of a typical cage rack. After completion of studies, animals were euthanized by CO_2_ followed by cervical dislocation. All test procedures were approved by Institutional Animal Care and Use Committee at the University of Texas Health Science Center at San Antonio.

### Blood glucose determination

Non-fasting blood glucose was measured after 4.5 weeks of age [6] during daylight hours (typically during the first half of the light cycle) using a blood glucose meter (AlphaTrak; Abbott Labs, Abbott Park, IL). Ins2^Akita^ mice develop hyperglycemia around 4 weeks of age, with a well-established sustained elevation of blood glucose and HbA_1c_ up until at least 9 months of age [6,7,27]. None of the Ctrl mice had glucose levels higher than 173 mg/dL; all Ins2^Akita^ mice had blood glucose values >250 mg/dL, a value which is generally considered to indicate diabetes in rodents [28]. Because at the age of testing many Ins2^Akita^ mice will have blood glucose levels above the highest level accurately detected by the meter (600 mg/dL), we did not perform correlations of any response values to blood glucose level.

### Electroretinogram (ERG)

Following over-night dark adaptation, animals were initially anesthetized with 4% isoflurane (Fluriso, VET One) and maintained at 1.5% delivered through a nose cone. Pupils were dilated with 1% tropicamide (Bausch + Lomb) and 2.5% phenylephrine (Paragon BioTeck). Eyes were anesthetized using 0.5% proparacaine (Apexa) and lubricated with 0.5% carboxymethylcellulose (Akorn). Binocular recordings were obtained using mouse ERG contact lens electrodes (LKC Technologies) with needle electrodes (OcuScience) in the neck and the tail serving as reference and ground, respectively. Dark-adapted, full-field ERGs were recorded (Espion ColorDome ganzfeld; Diagnosys, LLC) at a 1 kHz sampling rate and initially band-pass filtered at 0.3 to 300 Hz. Light stimuli consisted of 4 ms flashes ranging in scotopic (Sc) intensity from -4 to 0.8 log Sc Cd·s/m^2^. As flash intensities were increased, the inter-flash interval was also increased, ranging from 2 to 10 seconds, and the number of traces recorded to obtain the average response was decreased from 10 to 3 [29,30]. Analysis of individual traces within each stimulus intensity indicated that successive trials were similar in magnitude to initial ones. Each animal’s body temperature was maintained at 37˚C using a thermostatically controlled heating pad throughout the recordings, and all animals survived the procedure. For all animals, ERGs were recorded under two breathing conditions combined with isoflurane anesthesia at a flow rate of 2 liters/minute: 1) normal room air (i.e., normal atmosphere of 21% O_2_, 78% N_2_, 0.03% CO_2_ mixed with isoflurane to 1.2–1.5%) and 2) moderate hypercapnia (i.e., 5% CO_2_, 21% O_2_, 74% N_2_ (PraxAir, Inc.)) mixed with isoflurane to 1.2–1.5% in the same session. Isoflurane was used to provide consistency between the ERG and MRI recording conditions. Although different anesthetics can have differential effects on the ERG (isoflurane compared to ketamine and xylazine mixtures, for example), isoflurane is reported to be a useful, consistent alternative to other anesthetics for ERG recording [31]. Recordings under each breathing condition were approximately 30 minutes long with 15 minutes of breathing the second gas mixture occurring in the dark prior to initiation of the second set of ERG recordings. In both Diab and Ctrl groups, about half of the animals were initially tested under regular room air followed by hypercapnic condition and vice versa. Analysis of these two sub-groups showed that the order of the testing conditions did not significantly impact the outcomes. All animals underwent slit lamp examination after completion of ERG to observe for cataract and corneal haze. Although occasionally individual animals showed mild corneal haze (grade 1–2), no animal developed significant cataract [32].

### Choroidal and retinal blood flow determination using magnetic resonance imaging (MRI)

All animals underwent continuous arterial spin labeling (ASL) MRI to measure blood flow in the retina and choroid of the left eye within a week of ERG recordings. The ASL technique uses arterial blood water as an endogenous tracer by magnetically labeling the water of inflowing blood to a tissue, in this study at the heart/aorta, allowing for the calculation of volumetric blood flow pixel-by-pixel in units of ml of blood / g of tissue / min [33–35]. MRI scanning and analysis procedures of the retina followed methods described previously [9,36]. Animals were initially anesthetized with 4% isoflurane and maintained using 1.2–1.5% during scanning. Body temperature was maintained at 37°C. Respiratory rate was monitored with a pneumatic pressure sensor placed on the animal and was 80–110 breaths/min during anesthesia. The background light level during MRI was estimated to be very dim scotopic. We were unable to measure this directly due to incompatibility of the MR machine’s magnetic field with photometric equipment. However, ambient light was only from an observation window from a control room to the room with the scanner, the window was off-axis to the bore of the scanner, the mouse’s head was positioned approximately 75 cm deep into the bore with the mouse inserted head-first using a cylindrical holder 2 cm in diameter, and 55 cm of that bore has a diameter of 6 cm. In addition, the eye was surrounded by the coil during scanning. The mouse was wrapped in a paper towel as a blanket, and together with the mouse carrier and heating pad, these items also substantially block light access into the bore. As in the ERG recordings, MR scanning was performed under two breathing conditions, in normal room air and in moderate hypercapnic conditions, with both including isoflurane as the anesthetic agent.

MRI scans were performed in a 7T magnet (Biospec; Bruker, Billerica, MA), using custom eye and heart coils for MR imaging of blood flow using two-coil continuous ASL, with blood labeled at the heart and detected as it perfuses the retina and choroid [37]. MR parameters were as follows: gradient-echo echo planar imaging (EPI) sequence with field of view = 0.6x0.6 cm, matrix = 144x144, 2/3 partial Fourier acquisition, zero-filled interpolation to 256x256, 400 μm coronal slice, 2 shots, labeling duration = 2.6s, post label delay = 350ms, repetition time (TR) = 3s, and echo time (TE) = 9.8ms [9]. The imaging plane was positioned so as to bisect the eye into superior and inferior segments. Images were acquired with frequency encoding in the anteroposterior direction and with phase encoding in the left-right direction, which was approximately perpendicular to the nasal retina (see Fig 4). Blood flow MR imaging was performed for 5 min under air followed by 5 min under hypercapnia. After an interval of 5 min under air without scanning, blood flow MR imaging was performed again for 5 min under air and 5 min under hypercapnia. This timing was based on similar timeframes previously used in humans and rodents to measure the vascular reactivity and blood flow of the retinal and choroidal beds [16,38,39].

### Data analysis

**Fig 1:**
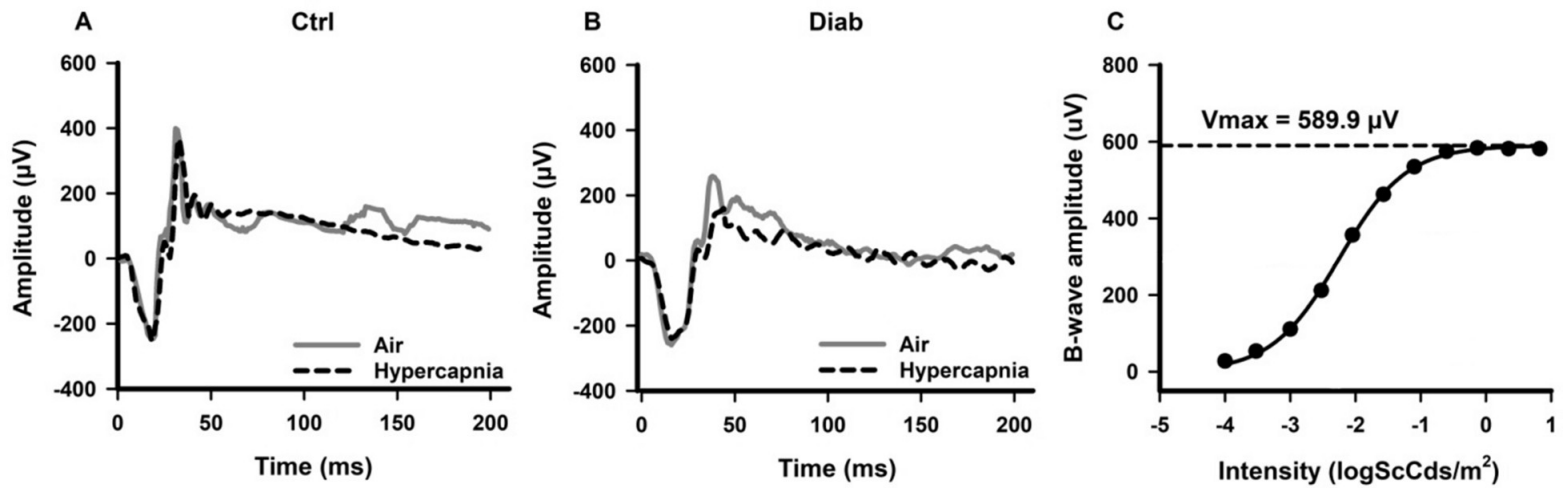
Examples of scotopic ERG waveforms recorded from a Ctrl mouse **(A)** and a Diab mouse **(B)** under regular room air condition (solid gray lines) and under hypercapnia (dashed black lines) after a short flash of -0.1 log Sc Cd·s/m^2^. An example of ERG b-wave intensity-response from a Ctrl mouse is shown with estimated Vmax from a Naka-Rushton fit **(C)**.

For both ERG and MRI, data from a single eye (left) of each animal was taken for analysis. ERG amplitudes and implicit times (IT) for the a-wave, b-wave, and OPs were calculated for all flash intensities. After OPs were removed by filtering (75 Hz low pass), a-wave amplitude was measured from baseline to the a-wave trough, and b-wave amplitude was measured from a-wave trough to b-wave peak. IT was measured from stimulus onset to respective trough or peak. For isolating OP responses for analysis, OPs were extracted with bandpass filtering from 75 to 300 Hz [8,40]. Amplitudes and ITs for five individual OPs (OP1 to OP5) were measured. Summed amplitude and IT for OPs were calculated [30]. Each animal’s b-wave intensity-response data were fit with a Naka-Rushton function to calculate Vmax (maximum amplitude) (Fig 1C).

For MRI, images were acquired as time series and averaged off-line using custom MATLAB routines. The retina was then flattened, motion correction was performed [36,41], and blood flow (BF) was calculated. The peak retinal BF (RBF) and choroidal BF (ChBF) were taken along the length of the retina and were averaged separately for the nasal and temporal regions [9]. The averaged regions of the retina extended from the edge of the optic nerve head to about 1.0–1.3 mm on each side (i.e., nasal and temporal) [42].

An *F*-test to compare variances between the two groups was initially performed. For initial analysis of ERG and MRI BF data, linear mixed models were used to test for significant effects due to group and breathing condition, including interaction term, and with the animal subject as a random factor (SPSS 27; IBM). For MRI BF data, the retinal region (nasal or temporal) was also included as a fixed factor, including interaction terms, to account for potential regional differences. A p < 0.05 was considered significant. Post-hoc tests were also performed. A two-tailed paired t-test was used to compare measures between air and hypercapnic conditions for each group of animals (Ctrl and Diab), and a two-tailed unpaired t-test (equal or unequal variances based on the *F*-tests) was used to compare measures between Ctrl and Diab groups for each breathing condition, air and hypercapnia (Excel, Microsoft). Following the advice of Armstrong 2014 [43], no corrections for multiple t-test comparisons were made to α-values; instead, p values greater than 0.01 but less than 0.05, which we interpret as marginally significant, are directly reported in Tables 2 and 3. Pearson’s correlation coefficient (r) was used to determine whether a significant relationship existed between ERG and BF measures.

## Results

The basic physiological parameters of both groups of mice (Ctrl and Diab) are shown in Table 1. Consistent with previous findings, Diab mice had significantly higher blood glucose levels and lower body weight than age-matched Ctrl animals.

**Table 1:** Physiological parameters in Ctrl and Diab animals (mean±SD).

	Ctrl	Diab
**Age, months**	8.3±0.5	8.2±0.6
**Weight, g**	32.0±3.5	23.7±1.4 ^**a**^
**Blood glucose, mg/dL**	150.3±38.2	528.8±71.0 ^**a**^

a: p<0.001 Diab vs. Ctrl.

### ERG responses while breathing regular room air revealed retinal deficits in Diab mice

We recorded ERGs while mice breathed regular room air. Example ERG responses to a scotopic flash from a Ctrl and a Diab mouse are shown in Fig 1A & 1B (solid traces, “Air” condition). The amplitudes and implicit times (IT) for the a-wave, b-wave, and OPs at stimulus intensity of -0.1 log Sc Cd·s/m^2^ as well as the Vmax parameter (an estimate of amplitude at saturation) of a Naka-Rushton fit of b-wave amplitude are summarized in Table 2 (mean ± SEM). An example Naka-Rushton fit of Ctrl data is shown in Fig 1C. From the initial linear mixed model analysis, there were significant differences between Diab and Ctrl, air and hypercapnia, and the group*hypercapnia interaction for the ERG b-wave Vmax, b-wave amplitude and IT, and the summed OPs amplitude and IT (p < 0.01), while there was a significant effect of hypercapnia on the a-wave amplitude (p = 0.029) and a significant group difference of the a-wave IT (p = 0.002).

**Table 2:** ERG measures recorded in regular room air and in hypercapnia (mean±SEM).

	Room Air		Hypercapnia	
	**Ctrl**	**Diab**	**Ctrl**	**Diab**
**Amplitude (μV)**				
**a-wave**	-218.5±16.6	-209.9±5.8	-203.5±7.7	-196.9±7.2 ^**d**^
**b-wave**	600.6±12.9	427.0±12.9 ^**a**^	592.4±11.2 ^**c1**^	351.1±14.0 ^**b**^^,^ ^**d**^
**b-wave (Vmax fit)**	600.1±13.9	422.2±10.7 ^**a**^	587.3±10.4 ^**c2**^	371.5±8.0 ^**b**^^,^ ^**d**^
**Summed OPs**	424.8±30.6	242.3±10.4 ^**a**^	433.9±28.5	168.9±10.3 ^**b**^^,^ ^**d**^
**Implicit time (ms)**				
**a-wave**	21.5±0.5	22.8±0.6	20.8±0.9	23.8±0.3 ^**b1**^
**b-wave**	32.0±0.5	37.2±0.4 ^**a**^	33.1±1.2	42.8±0.5 ^**b**^^,^ ^**d**^
**Summed OPs**	147.3±5.6	165.5±1.8 ^**a**^	151.8±5.3	181.6±1.5 ^**b**^^,^ ^**d**^

Amplitudes and implicit times are from -0.1 log Sc Cd·s/m^2^ stimulus intensity.

a: p<0.01 for Ctrl vs. Diab in room air.

b: p<0.01 for Ctrl vs. Diab in hypercapnia

b1: p = 0.014.

c1: p = 0.026

c2: p = 0.044 for Ctrl room air vs. Ctrl hypercapnia.

d: p<0.01 for Diab room air vs. Diab hypercapnia.

While breathing room air, the inner retinal ERG responses were compromised in Diab animals. At a stimulus intensity of -0.1 log Sc Cd·s/m^2^, which elicited maximal responses in Ctrl mice, both b-wave and OP responses showed significant reduction in amplitudes and delays in IT in Diab (b-wave amplitudes: 427.0 ± 12.9 μV in Diab vs 600.6 ± 12.9 in Ctrl, p < 0.001, b-wave IT: 37.2 ± 0.4 ms in Diab vs 32.0 ± 0.5 ms in Ctrl, p < 0.001). The Vmax from Naka-Rushton fits to the b-wave data was also significantly reduced in Diab compared to Ctrl mice (422.2 ± 10.7 μV in Diab vs. 600.1 ± 13.9 μV in Ctrl, p < 0.001). Although a-wave responses appeared slightly reduced and delayed in Diab, they did not differ significantly from Ctrl mice under air (p > 0.1) (Table 2).

### ERG responses during moderate hypercapnia revealed additional retinal deficits in Diab mice

**Fig 2:**
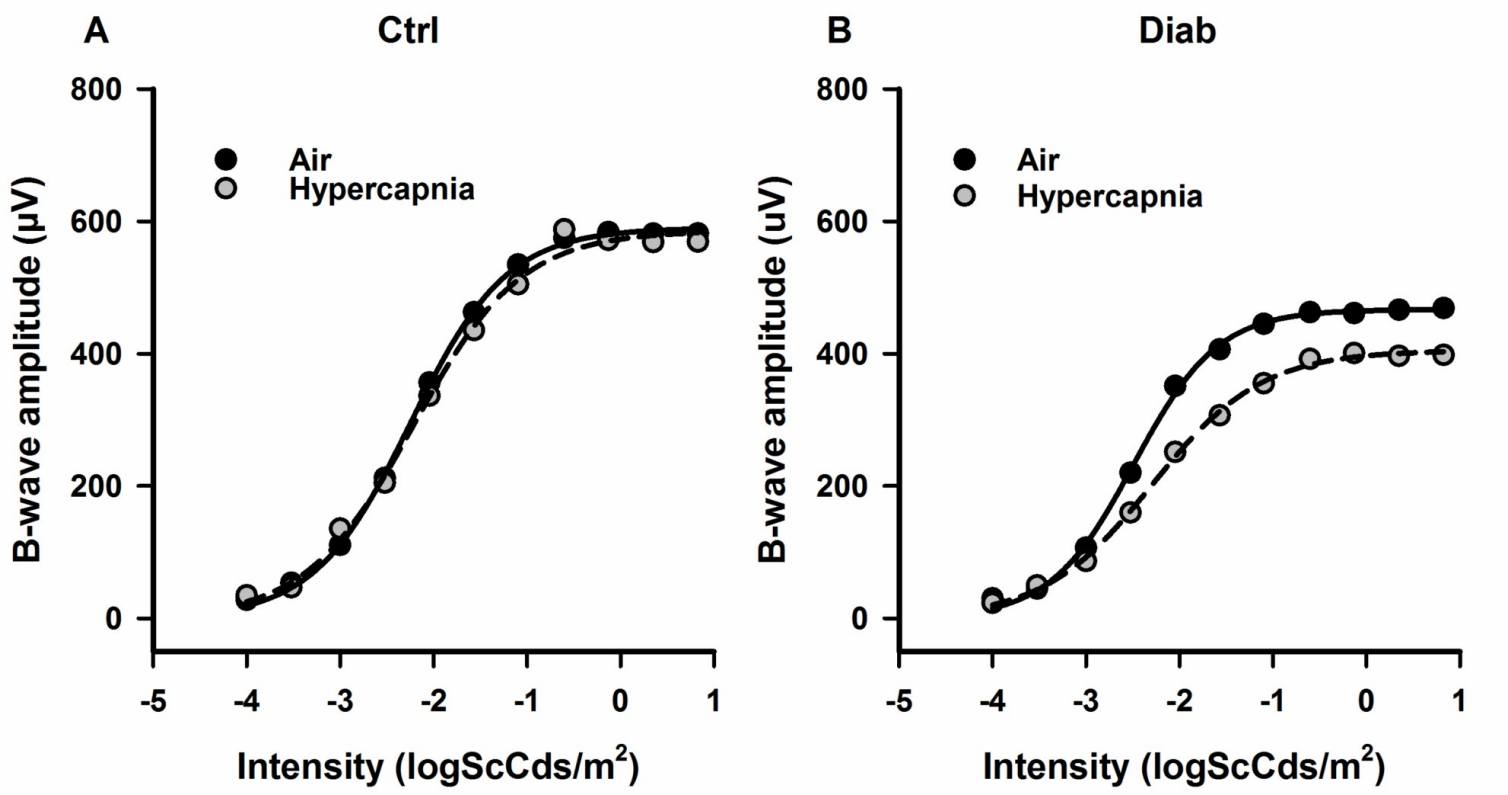
Examples of ERG intensity-response series for b-wave amplitudes fit with a Naka-Rushton function from a Ctrl mouse **(A)** and Diab mouse **(B)** under regular room air condition (black circles with solid line fit) and under hypercapnia (gray circles with dashed line fit). (Group means are provided in Table 2).

**Fig 3:**
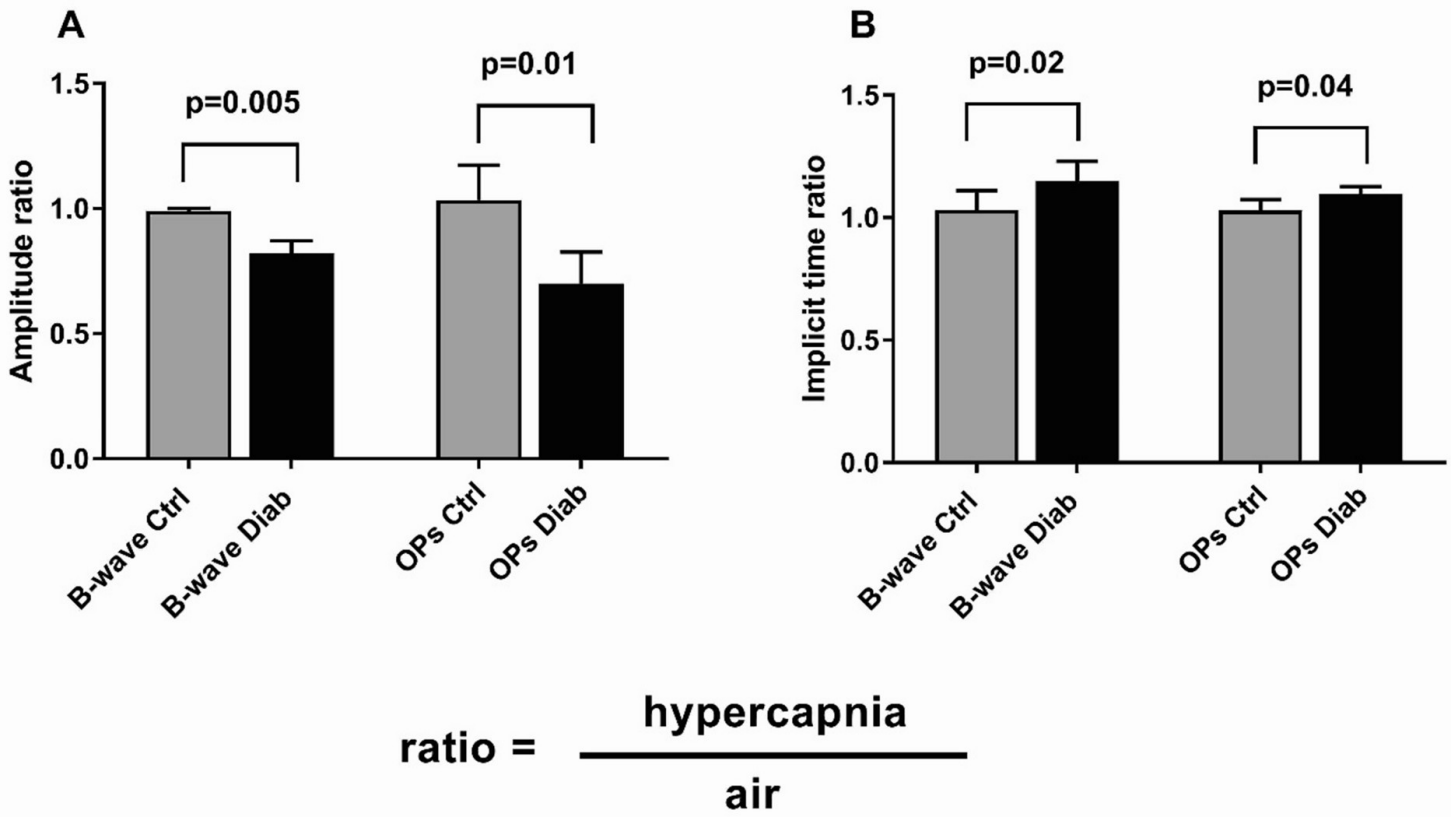
Ratios of hypercapnia to air values of ERG b-wave and OP amplitudes **(A)** and implicit times **(B)** among Ctrl and Diab groups for a stimulus intensity of -0.1 log Sc Cd·s/m^2^. Error bars show standard errors.

To determine whether Diab mice have an impaired response compared to Ctrl mice during a metabolic challenge, we also recorded ERGs from Ctrl and Diab mice using the same stimulus parameters but while the mice breathed 5% CO_2_, a moderate hypercapnic challenge. Example ERG responses during air and hypercapnia from individual Ctrl and Diab mice are shown in Fig 1A & 1B (*dashed* traces, hypercapnia condition). The results are summarized in Table 2. During hypercapnia in Ctrl mice, b-wave Vmax responses were only slightly reduced (by 2.0 ± 0.8%; p = 0.044) when compared to responses in room air. However, Diab mice showed further compromise (by 11.8 ± 2.0%; p = 0.001) in b-wave Vmax response during hypercapnia (Table 2 and Fig 2A and 2B). As shown in Fig 3, in which ERG values during hypercapnia are normalized to responses during air breathing, in Diab mice, the b-wave and OP amplitudes were significantly worse during hypercapnic challenge compared to values obtained under regular room air (for example, 17.9 ± 1.4% reduction in b-wave amplitude during hypercapnia in Diab (p < 0.001) vs a 1.3 ± 0.5% reduction in Ctrl (p = 0.026)). Similarly, both b-wave and OP implicit times were further delayed in Diab mice during hypercapnia (15.2 ± 1.8% longer delay in b-wave IT in Diab (p < 0.001) vs insignificant 3.1 ± 2.3% longer delay in Ctrl (p = 0.20)). Ctrl mice did not show any significant change in a-wave amplitude or IT during hypercapnia (p > 0.05), suggesting that hypercapnia did not impair outer retinal function. However, while Diab mice had no change in a-wave IT under hypercapnia (p = 0.17), their mean a-wave amplitude was significantly reduced by hypercapnia (by 6.3 ± 1.8%; p = 0.009).

### MRI while breathing regular room air showed reduced choroidal and retinal blood flow in Diab mice

**Fig 4:**
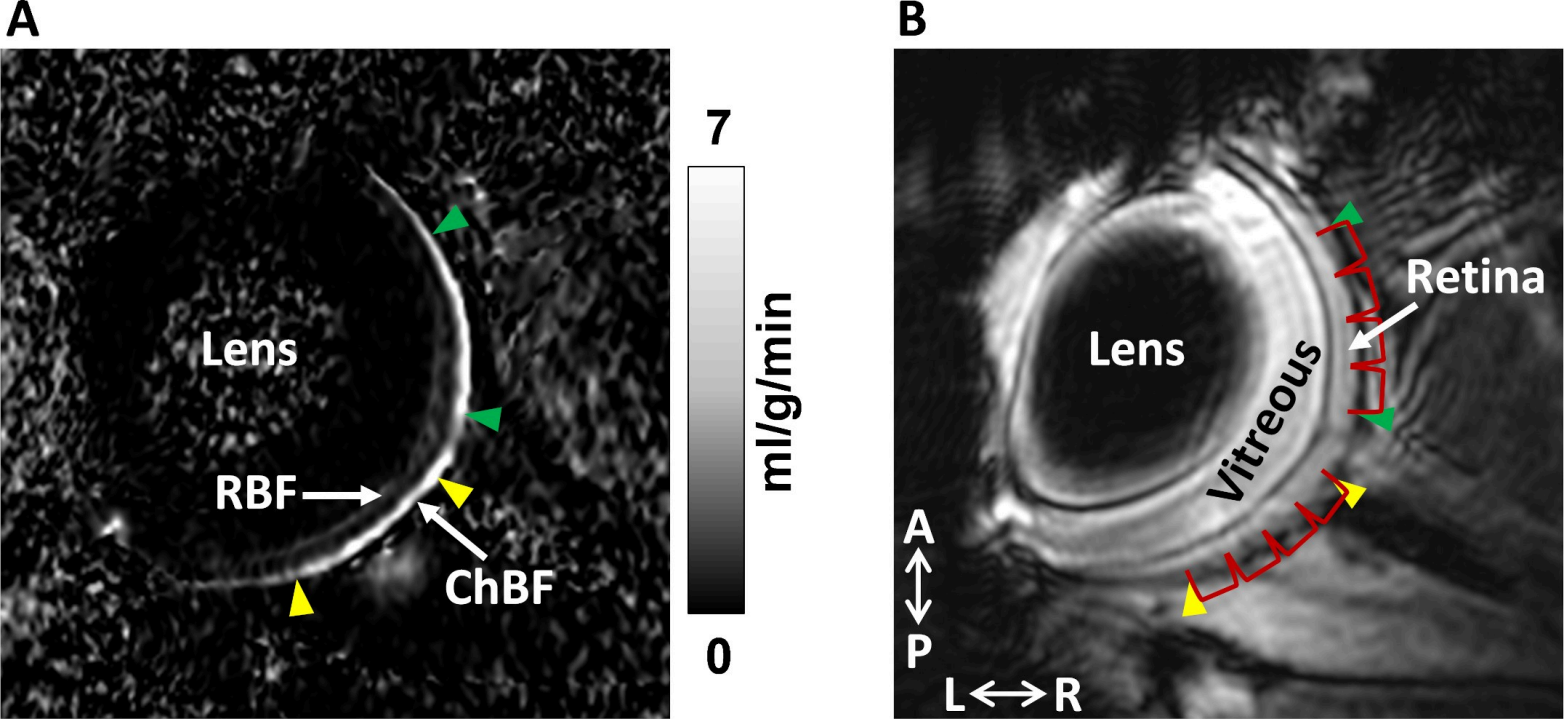
Example MRI blood flow map of a mouse eye **(A)** and corresponding anatomical EPI image **(B)**. Retinal blood flow (RBF) and choroidal blood flow (ChBF) are indicated. The regions from which blood flow were measured are indicated as the regions between the two green (nasal) or the two yellow (temporal) arrowheads. The four bins from both regions from which data were averaged over for plotting in Fig 5 are indicated by red brackets. A: anterior, P: posterior, L: left, R: right. The blood flow map is scaled from 0 to 7 ml/g/min.

**Fig 5:**
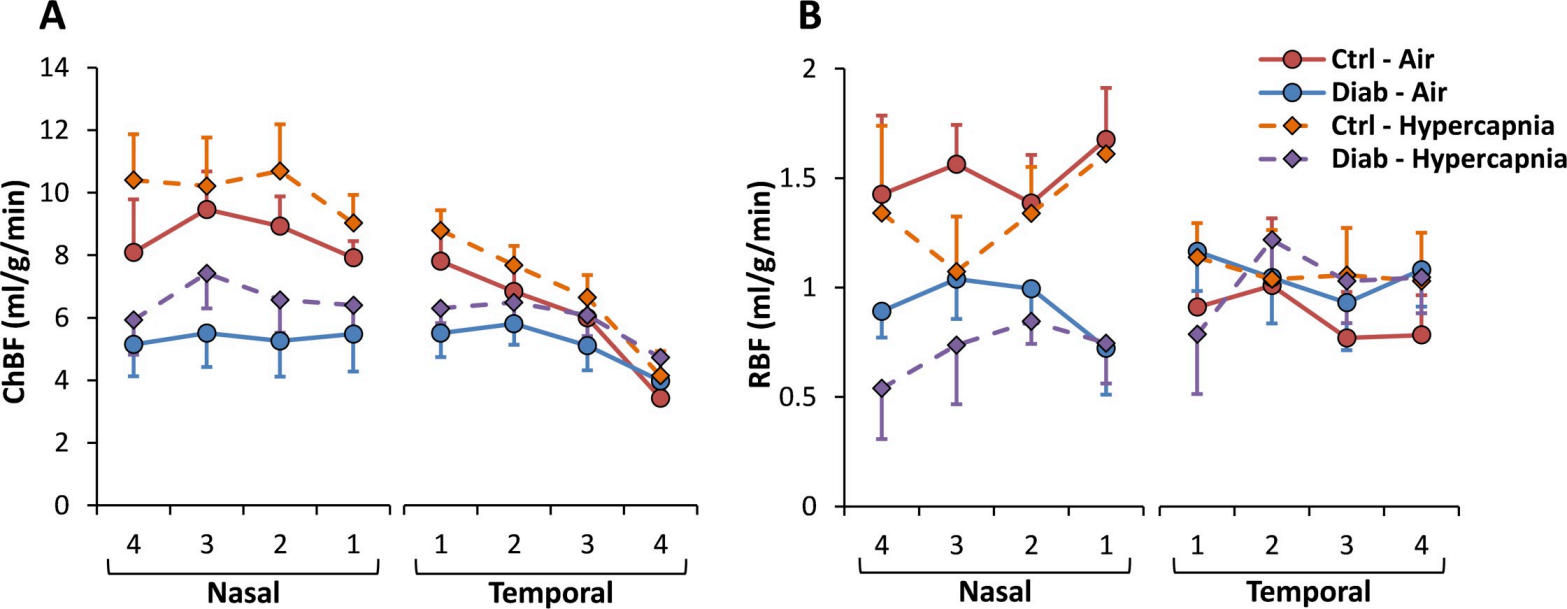
Choroidal blood flow (ChBF) **(A)** and retinal blood flow (RBF) **(B)** plotted along the length of the retina for Ctrl and Diab mice under air and hypercapnia. Blood flow data from each retinal region (nasal or temporal), which were taken pixel-by-pixel, were averaged over four bins in each region for this plot. Error bars show standard errors.

Example MRI images and blood flow map are shown from an individual mouse in Fig 4. Group-mean ChBF and RBF under air and hypercapnia are plotted along the length of the retina in Fig 5. From this data, both RBF and ChBF appeared larger and showed large group differences in the nasal region, while the BF in the temporal region tended to be lower with little group difference, which could obscure the regional group difference if averaged together. Initial statistical analysis of these apparent regional differences, using a linear mixed model analysis including region as a factor, supported this difference, with the region having a significant effect in ChBF (p < 0.001). The group*region interaction term also had a significant effect for both ChBF (p = 0.002) and RBF (p < 0.001), indicating regional group differences. Hypercapnia had a significant effect of increasing ChBF (p = 0.005), with no significant interaction between group and the hypercapnia response for either ChBF or RBF (p > 0.05). This regional difference could arise from physiological differences between regions or due to technical factors from the MRI acquisition which may mask some temporal blood flow in the MRI sequence used here (see Discussion). Given these regional differences, further BF data presented and analyzed here used only the average nasal BF. ChBF and RBF from the nasal region in Ctrl and Diab mice breathing regular room air and under hypercapnia are given in Table 3 (mean ± SEM). While breathing room air, ChBF was reduced in Diab when compared to Ctrl mice by 38% (5.4 ± 1.0 ml/g/min in Diab vs. 8.6 ± 1.0 ml/g/min in Ctrl, p = 0.048). RBF was reduced in Diab by 40% (0.91 ± 0.10 ml/g/min in Diab vs. 1.52 ± 0.24 ml/g/min in Ctrl, p = 0.037).

**Table 3:** MRI BF measures from the nasal region recorded in regular room air and in hypercapnia (mean±SEM).

	Room Air		Hypercapnia	
	Ctrl	Diab	Ctrl	Diab
**ChBF (ml/g/min)**	8.6±1.0	5.4±1.0 ^**a1**^	10.1±1.2 ^**c1**^	6.6±1.0 ^**b1**^^,^ ^**d**^
**RBF (ml/g/min)**	1.52±0.24	0.91±0.10 ^**a2**^	1.35±0.20	0.72±0.09 ^**b2**^

a1: p = 0.048

a2: p = 0.037 for Ctrl vs. Diab in room air.

b1: p = 0.049

b2: p = 0.014 for Ctrl vs. Diab in hypercapnia.

c1: p = 0.029 for Ctrl room air vs. Ctrl hypercapnia.

d: p<0.01 for Diab room air vs. Diab hypercapnia.

### Choroidal blood flow increased during moderate hypercapnia in Ctrl and Diab mice

**Fig 6:**
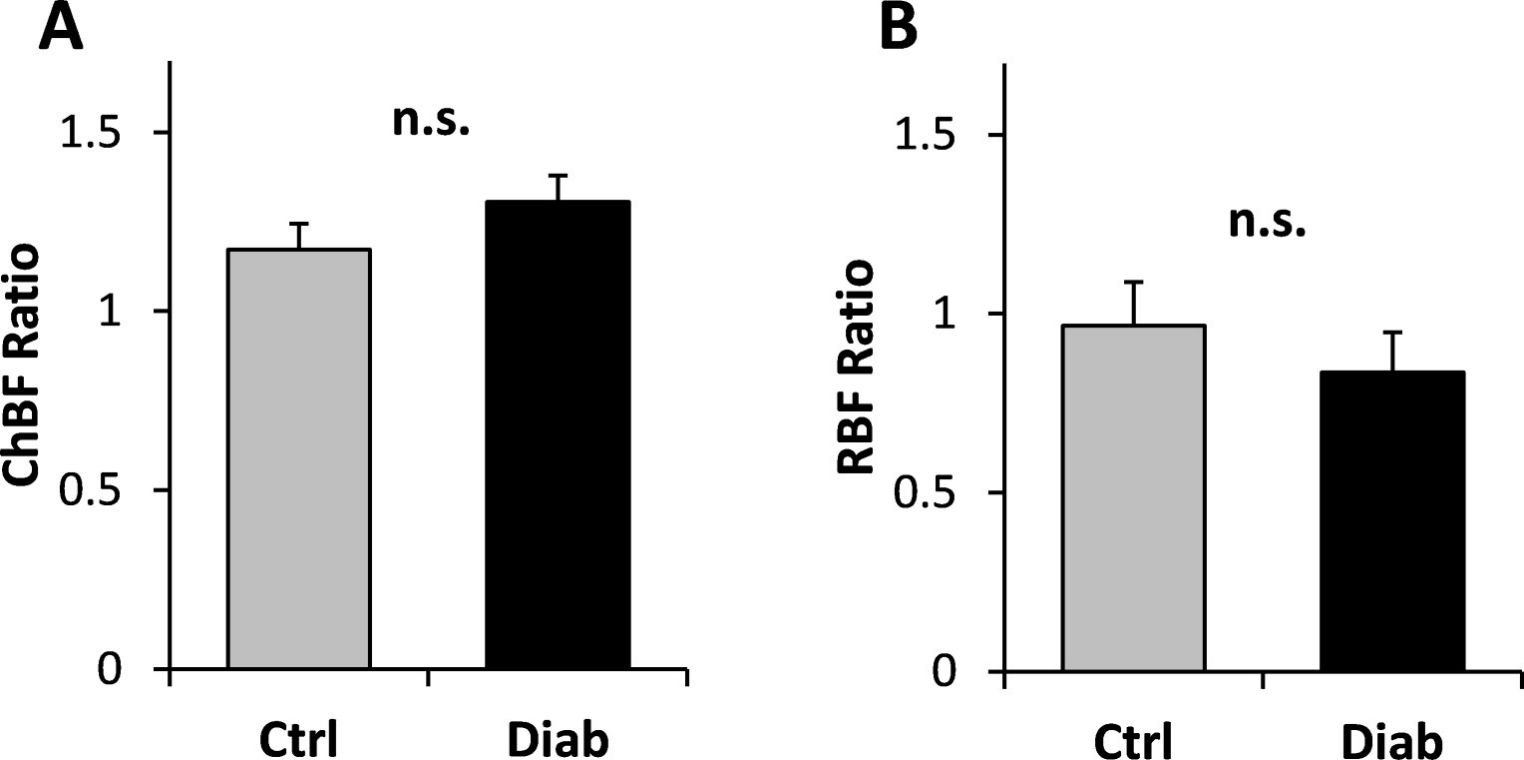
Ratios of hypercapnia to air values of choroidal blood flow (ChBF) **(A)** and retinal blood flow (RBF) **(B)** from the nasal region for Ctrl and Diab mice determined using MRI. Ratios were not significantly different between Ctrl and Diab (n.s.). Error bars show standard errors.

To determine if diabetic Ins2^Akita^ mice can increase blood flow similar to non-diabetic mice during the metabolic challenge, we determined ocular blood flow using MRI during hypercapnia. Both Ctrl and Diab groups had significantly increased ChBF in the nasal regions during hypercapnia (Table 3). Under hypercapnia, ChBF and RBF remained lower in Diab compared to Ctrl mice (ChBF: 6.6 ± 1.0 ml/g/min in Diab vs. 10.1 ± 1.2 ml/g/min in Ctrl, p = 0.049; RBF: 0.72 ± 0.09 ml/g/min in Diab vs. 1.35 ± 0.20 ml/g/min in Ctrl, p = 0.014). As shown by blood flow values during hypercapnia normalized to the regular room air value (Fig 6A), ChBF increased by 17 ± 7% in Ctrl (p = 0.029) and by 31 ± 7% in Diab (p = 0.006). The extent of the increase in ChBF was not significantly different between Diab and Ctrl groups (p = 0.22). A significant change in RBF due to hypercapnia was not detected in either group (-3 ± 12% in Ctrl and -16 ± 11% in Diab, p > 0.05 for both), and the RBF change was not significantly different between the two groups (p = 0.45) (Fig 6B; see Discussion).

### Correlation of b-wave and blood flow deficits in Diab and Ctrl mice

**Fig 7:**
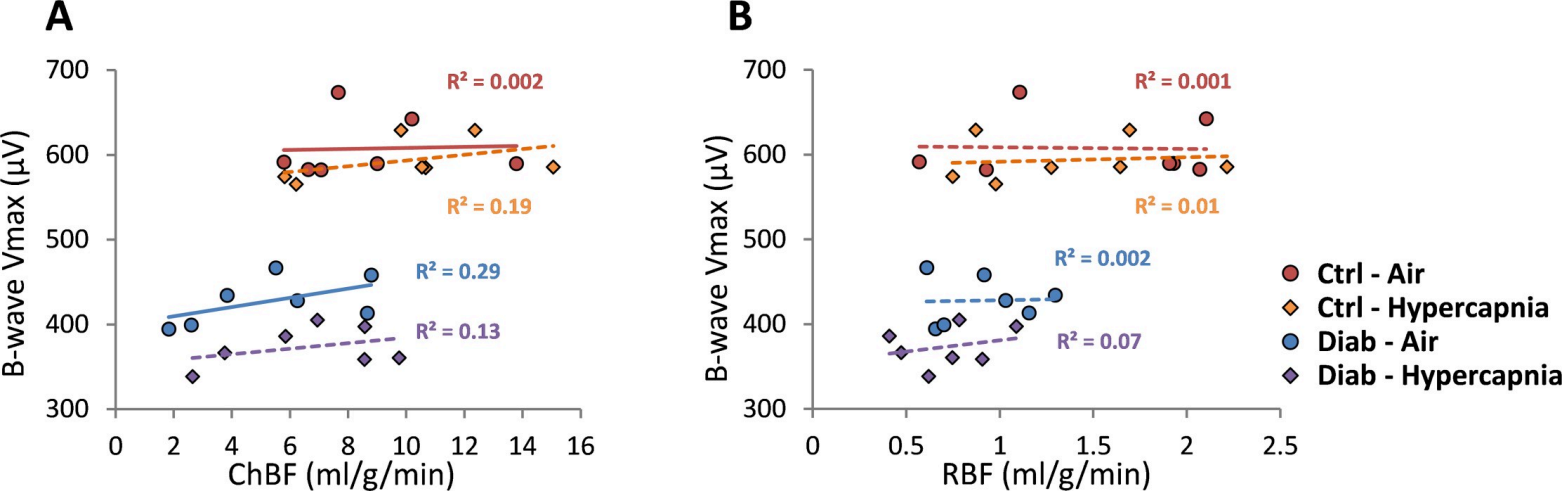
Correlations between ERG b-wave Vmax with ChBF **(A)** or RBF **(B)** from the nasal region under regular room air and under hypercapnia for Ctrl and Diab groups. The straight lines are fitted linear regression lines with R^2^ values shown.

To probe the relationship between blood flow and retinal neuronal performance in Ctrl and Diab mouse eyes, we performed correlation analysis between ChBF and RBF with the ERG b-wave Vmax parameters (Fig 7). For both Ctrl and Diab in both breathing conditions (i.e., room air and hypercapnia), neither ChBF nor RBF showed significant correlation with ERG b-wave Vmax (p > 0.05). The ChBF had a trend of weak to moderate correlation in Diab and under hypercapnia (r = 0.36 to 0.54), while RBF had low correlation with b-wave Vmax for all groups and conditions (r = 0.03 to 0.27).

## Discussion

We found that diabetic Ins2^Akita^ mice after 6–7 months of diabetes have reduced ERG b-wave and OP amplitudes and delayed b-wave and OP implicit times compared to age-matched, non-diabetic Ctrl mice, when breathing normal air. ChBF and RBF were also significantly reduced in Diab mice. However, neither ChBF nor RBF were significantly correlated with ERG b-wave Vmax in either Ctrl or Diab mice. Diabetes may disrupt the blood flow response of the retina to metabolic challenges due to vascular cell injury, including injury to or loss of endothelial cells and pericytes [3]. Thus, we further tested the ERG and ocular blood flow responses to metabolic challenge using moderate hypercapnic conditions (breathing 5% CO_2_ mixed with room air). Ctrl mice had only slight reduction of the b-wave amplitude and otherwise maintained their ERG performance under hypercapnia, and their ChBF significantly increased. In Diab mice, hypercapnia led to further deficits in all components of the ERG, while the increase in ChBF was not significantly affected compared to Ctrl mice.

To examine whether the ERG deficits during moderate hypercapnia in the diabetic retina are associated with the insufficiency of blood flow or blood flow response to hypercapnia, we examined correlations between ERG parameters and ChBF and RBF. The ERG changes were predominantly inner retinal because the b-wave and OP amplitudes were reduced. A previous study using Ins2^Akita^ mice reported a significantly reduced scotopic b-wave amplitude at 9 but not 6 months of age [7]. The a-wave amplitude was also reported to be reduced at 9 months of age but not 6 months [7], so it is possible the small changes we found in the a-wave response to hypercapnia would become greater in older mice. While DR is traditionally considered a vascular disease, neuronal damage may occur in the retina directly through factors such as hyperglycemia or hypoinsulemia [3], so it may be that the RBF, ChBF, and ERG b-wave/OPs are independently affected by systemic factors and thus were not significantly correlated.

Retinal physiology and metabolism can be modulated in several ways, with various effects on function of the healthy and diabetic retina. Breathing pure oxygen to increase blood oxygenation has been shown to improve vision and the ERG responses in diabetic patients [44,45], so increasing ocular blood flow may also be expected to improve retinal function. While experimental manipulations that affect blood flow can also affect ERG performance, the direct functional connection remains unresolved. Retinal ischemia, which decreases ocular blood flow, leads to significantly decreased ERG b-wave amplitude in normal rats, and reperfusion after ischemia leads to recovery of the b-wave amplitude [46]. On the other hand, when normal or diabetic rodents are treated with the nitric oxide synthase (NOS) inhibitor L-nitro-arginine methyl ester (L-NAME), which decreases ocular blood flow but less severely than experimental ischemia, the ERG remains unaffected [47–50].

Although hypercapnia increases ocular blood flow, it has been shown to significantly decrease the ERG b-wave amplitude in normal cats [22,24] and dogs [25], likely due to plasma, and thus retinal, acidosis. Ctrl mice here had only a small reduction of the b-wave amplitude during hypercapnia. This could be due to differences in species, anesthesia, or severity of hypercapnia. The Diab mice, however, had further ERG b-wave deficits under hypercapnia, supporting the hypothesis that the diseased retinas are more sensitive to hypercapnic changes. This finding is consistent with a study showing that hypercapnia decreased contrast sensitivity in glaucoma patients but not in healthy humans [26]. It also remains unclear if the potential to buffer or compensate for retinal acidotic changes is compromised in the visual system in diabetes. A recent study using the streptozotocin-induced diabetic rat showed that diabetes itself induces retinal acidosis, but this was only consistently observed during early stages of the disease, while after 3 months of diabetes the retinal pH became more variable relative to healthy eyes, possibly due to compensations that improve acid handling by the retina [51]. To address this, future experiments could be aimed at inducing metabolic acidosis by dietary modifications such as addition of ammonium chloride to drinking water [52] and longitudinally tracking neuronal and vascular effects of chronic acidosis independent of hypercapnia in healthy and diabetic eyes.

We have previously found that both ChBF and RBF are reduced in Diab mice compared to Ctrl, with the ChBF deficit preceding functional optokinetic deficits [9], and other studies have found reduced RBF in diabetic Ins2^Akita^ mice using optical imaging methods [53,54]. RBF and ChBF in nasal and temporal regions were significantly different and with large group differences only detected in the nasal region. These regional BF differences could potentially arise from biological differences between regions and technical factors with the MRI protocol. The retinal vasculature is different in nasal and temporal quadrants in foveated retinas, so RBF might be expected to vary between regions, but mice lack a fovea, so it is unclear whether they would have nasal-temporal BF differences in the retina or choroid. Technical factors include the artifacts associated with EPI sequences which can affect the phase and frequency encoding directions differently, such as low relative bandwidth and geometric distortion with phase encoding or non-uniform ramp-sampling with frequency encoding. EPI is sensitive to magnetic inhomogeneity, which is relatively large in the orbit due to nearby air-tissue interfaces and potentially from the surrounding large veins and fat in the orbit, which could lead to spatially-localized artifacts. Future studies could explore alternative MRI sequences to investigate the contribution of biological and technical factors to the regional differences found herein. For example, turbo spin echo, which we have used for human ocular BF imaging [55,56], is not susceptible to these spatial artifacts, albeit the signal to noise ratio can be notably lower, affecting the ability to detect the normally low RBF. Other factors could have affected MRI measurements of ocular blood flow. The reduced weight and body fat of the Diab mice could potentially have affected labeling efficiency for ASL, but this would reduce the depth to the heart in Diab which would be expected to improve labeling efficiency, giving higher blood flow values. However, labeling efficiency was likely unaffected since the two-coil continuous ASL used velocity-driven adiabatic inversion, which is insensitive to RF amplitude [57,58]. Isoflurane is a vasodilator which dose-dependently increases blood flow [59] and which may be more potent in diabetes [60]. A higher sensitivity of Diab mice to isoflurane could possibly cause a larger increase in basal blood flow relative to Ctrl mice; however, ChBF and RBF were still significantly reduced in Diab mice in the nasal region, suggesting the effects of anesthesia did not substantially affect the blood flow outcomes between groups. It is not clear why RBF is not affected by hypercapnia while the ChBF increased. It is possible that the relatively low basal blood flow signal in the retinal circulation made detection of blood flow changes more difficult. Anesthetic effects of vasodilatory isoflurane could be another reason [61]. Human studies did not use anesthesia, with RBF reported to increase due to hypercapnia [62] but to decrease while breathing carbogen [17]. Previous animal studies used different preparations, chloralose anesthesia (vasoconstrictive) or mechanically-ventilated isoflurane anesthesia, or different hemodynamic measurements (blood volume) [15,18]. Studies under similar conditions but with other methods to measure RBF or with other anesthetics are needed. In non-diabetic C57BL6 mice, moderate hypercapnia does not significantly affect heart rate and significantly increases systolic blood pressure but only by a few percent [63,64]. Diabetic Ins2^Akita^ mice on the C57BL6 background breathing normal air have been reported to have a higher systolic blood pressure than non-diabetic mice, ~9% at 6 months of age [65], but have also been reported to have normal systolic function combined with diastolic dysfunction at 3 and 6 months of age [66]. Although we did not monitor blood pressure and heart rate during recordings, isoflurane anesthesia at the level used here is expected to have very little if any effect on these parameters in the C57BL6 mouse [67]. Thus, while the published blood pressure response to hypercapnia is quite consistent and relatively small across several strains of non-diabetic mice, the blood pressure response of the diabetic Ins2^Akita^ mouse to hypercapnia remains unknown. While several confounding factors potentially affect ocular blood flow measurements in diabetic animals, these findings in Diab mice suggest that future studies in human diabetic patients should include not only measurements of blood flow and regulation in the retina but in the choroid as well.

In summary, we found that diabetic Ins2^Akita^ mice have significant deficits in retinal neuronal function and choroidal and retinal blood flow and show further compromise in retinal neuronal function, but not blood flow, when exposed to the metabolic challenge of moderate hypercapnia. While DR is traditionally considered a vascular disease, we did not find significant correlation between retinal neuronal function with retinal or choroidal blood flow, suggesting that neuronal and vascular damage may occur independently in the retina directly through systemic factors such as hyperglycemia or hypoinsulemia. Our results support the idea that the diabetic retina has difficulty adapting to metabolic challenges due to factors other than impaired blood flow regulation.

## Supporting information

S1 Table Data from individual animals. All physiology, ERG, and blood flow data are given.
(XLSX)

## References

[1] Feit-Leichman RA, Kinouchi R, Takeda M, Fan Z, Mohr S, Kern TS, et al. Vascular damage in a mouse model of diabetic retinopathy: relation to neuronal and glial changes. Invest Ophthalmol Vis Sci. 2005;46(11):4281–7. doi: 10.1167/iovs.04-1361 . 16249509

[2] Kur J, Newman EA, Chan-Ling T. Cellular and physiological mechanisms underlying blood flow regulation in the retina and choroid in health and disease. Prog Retin Eye Res. 2012;31(5):377–406. doi: 10.1016/j.preteyeres.2012.04.004 . 22580107 PMC3418965

[3] Abcouwer SF, Gardner TW. Diabetic retinopathy: loss of neuroretinal adaptation to the diabetic metabolic environment. Ann N Y Acad Sci. 2014;1311:174–90. doi: 10.1111/nyas.12412 . 24673341 PMC4154702

[4] Eisma JH, Dulle JE, Fort PE. Current knowledge on diabetic retinopathy from human donor tissues. World J Diabetes. 2015;6(2):312–20. doi: 10.4239/wjd.v6.i2.312 . 25789112 PMC4360424

[5] Barber AJ, Lieth E, Khin SA, Antonetti DA, Buchanan AG, Gardner TW. Neural apoptosis in the retina during experimental and human diabetes. Early onset and effect of insulin. J Clin Invest. 1998;102(4):783–91. doi: 10.1172/JCI2425 9710447 PMC508941

[6] Barber AJ, Antonetti DA, Kern TS, Reiter CE, Soans RS, Krady JK, et al. The Ins2Akita mouse as a model of early retinal complications in diabetes. Invest Ophthalmol Vis Sci. 2005;46(6):2210–8. doi: 10.1167/iovs.04-1340 . 15914643

[7] Han Z, Guo J, Conley SM, Naash MI. Retinal angiogenesis in the Ins2(Akita) mouse model of diabetic retinopathy. Invest Ophthalmol Vis Sci. 2013;54(1):574–84. doi: 10.1167/iovs.12-10959 . 23221078 PMC3558298

[8] Hombrebueno JR, Chen M, Penalva RG, Xu H. Loss of synaptic connectivity, particularly in second order neurons is a key feature of diabetic retinal neuropathy in the Ins2Akita mouse. PLoS One. 2014;9(5):e97970. doi: 10.1371/journal.pone.0097970 . 24848689 PMC4029784

[9] Muir ER, Renteria RC, Duong TQ. Reduced ocular blood flow as an early indicator of diabetic retinopathy in a mouse model of diabetes. Invest Ophthalmol Vis Sci. 2012;53(10):6488–94. doi: 10.1167/iovs.12-9758 . 22915034 PMC4045095

[10] Pournaras CJ, Rungger-Brandle E, Riva CE, Hardarson SH, Stefansson E. Regulation of retinal blood flow in health and disease. Prog Retin Eye Res. 2008;27(3):284–330. doi: 10.1016/j.preteyeres.2008.02.002 . 18448380

[11] Cioffi GA, Granstam E, Alm A. Ocular Circulation. In: Kaufman PL, Alm A, editors. Adler’s Physiology of the Eye - 10th ed. St. Louis: Mosby; 2003. p. 747–76.

[12] Riva CE, Grunwald JE, Singclair SH. Laser Doppler velocimetry study of the effect of pure oxygen breathing on retinal blood flow. Invest Ophthalmol Vis Sci. 1983;24:47–51. 6826314

[13] Geiser MH, Riva CE, Dorner GT, Diermann U, Luksch A, Schmetterer L. Response of choroidal blood flow in the foveal region to hyperoxia and hyperoxia-hypercapnia. Curr Eye Res. 2000;21(2):669–76. . 11148604

[14] Riva CE, Cranstoun SD, Grunwald JE, Petrig BL. Choroidal blood flow in the foveal region of the human ocular fundus. Invest Ophthalmol Vis Sci. 1994;35:4273–81. 8002247

[15] Alm A, Bill A. The oxygen supply to the retina: II. Effects of high intraocular pressure and of increased arterial carbon dioxide tension on uveal and retinal blood flow in cats. Acta Physiol Scand. 1972;84:306–19. doi: 10.1111/j.1748-1716.1972.tb05182.x 4553229

[16] Wang L, Grant C, Fortune B, Cioffi GA. Retinal and choroidal vasoreactivity to altered PaCO2 in rat measured with a modified microsphere technique. Exp Eye Res. 2008;86(6):908–13. doi: 10.1016/j.exer.2008.03.005 . 18420196

[17] Pakola SJ, Grunwald JE. Effects of oxygen and carbon dioxide on human retinal circulation. Invest Ophthalmol Vis Sci. 1993;34(10):2866–70. . 8360019

[18] Nair G, Tanaka Y, Kim M, Olson DE, Thule PM, Pardue MT, et al. MRI reveals differential regulation of retinal and choroidal blood volumes in rat retina. Neuroimage. 2011;54(2):1063–9. doi: 10.1016/j.neuroimage.2010.09.020 . 20850550 PMC3190232

[19] Berkowitz BA, Kowluru RA, Frank RN, Kern TS, Hohman TC, Prakash M. Subnormal retinal oxygenation response precedes diabetic-like retinopathy. Invest Ophthalmol Vis Sci. 1999;40(9):2100–5. . 10440266

[20] Ashimatey BS, Green KM, Chu Z, Wang RK, Kashani AH. Impaired Retinal Vascular Reactivity in Diabetic Retinopathy as Assessed by Optical Coherence Tomography Angiography. Invest Ophthalmol Vis Sci. 2019;60(7):2468–73. doi: 10.1167/iovs.18-26417 . 31173077 PMC6557617

[21] Andersen MV. Changes in the vitreous body pH of pigs after retinal xenon photocoagulation. Acta Ophthalmol (Copenh). 1991;69(2):193–9. doi: 10.1111/j.1755-3768.1991.tb02710.x 1908170

[22] Niemeyer G, Steinberg RH. Differential effects of pCO2 and pH on the ERG and light peak of the perfused cat eye. Vision Res. 1984;24(3):275–80. doi: 10.1016/0042-6989(84)90131-7 6426165

[23] Hiroi K, Yamamoto F, Honda Y. Analysis of electroretinogram during systemic hypercapnia with intraretinal K(+)-microelectrodes in cats. Invest Ophthalmol Vis Sci. 1994;35(11):3957–61. . 7928195

[24] Linsenmeier RA, Mines AH, Steinberg RH. Effects of hypoxia and hypercapnia on the light peak and electroretinogram of the cat. Invest Ophthalmol Vis Sci. 1983;24(1):37–46. . 6826313

[25] Varela Lopez O, Alvarez Vazquez JC, Gonzalez Cantalapiedra A, Rosolen SG. Effects of hypercapnia on the electroretinogram in sevoflurane and isoflurane anaesthetized dogs. Doc Ophthalmol. 2010;121(1):9–20. doi: 10.1007/s10633-010-9223-4 . 20145987

[26] Hosking SL, Evans DW, Embleton SJ, Houde B, Amos JF, Bartlett JD. Hypercapnia invokes an acute loss of contrast sensitivity in untreated glaucoma patients. Br J Ophthalmol. 2001;85(11):1352–6. doi: 10.1136/bjo.85.11.1352 . 11673305 PMC1723756

[27] Naito M, Fujikura J, Ebihara K, Miyanaga F, Yokoi H, Kusakabe T, et al. Therapeutic impact of leptin on diabetes, diabetic complications, and longevity in insulin-deficient diabetic mice. Diabetes. 2011;60(9):2265–73. doi: 10.2337/db10-1795 . 21810600 PMC3161331

[28] Clee SM, Attie AD. The genetic landscape of type 2 diabetes in mice. Endocr Rev. 2007;28(1):48–83. doi: 10.1210/er.2006-0035 . 17018838

[29] Wang J, Mojumder DK, Yan J, Xie A, Standaert RF, Qian H, et al. In vivo electroretinographic studies of the role of GABAC receptors in retinal signal processing. Exp Eye Res. 2015;139:48–63. doi: 10.1016/j.exer.2015.07.002 . 26164072 PMC4573340

[30] Mocko JA, Kim M, Faulkner AE, Cao Y, Ciavatta VT, Pardue MT. Effects of subretinal electrical stimulation in mer-KO mice. Invest Ophthalmol Vis Sci. 2011;52(7):4223–30. doi: 10.1167/iovs.10-6750 . 21467171 PMC3175956

[31] Woodward WR, Choi D, Grose J, Malmin B, Hurst S, Pang J, et al. Isoflurane is an effective alternative to ketamine/xylazine/acepromazine as an anesthetic agent for the mouse electroretinogram. Doc Ophthalmol. 2007;115(3):187–201. doi: 10.1007/s10633-007-9079-4 . 17885776

[32] Wolf NS, Li Y, Pendergrass W, Schmeider C, Turturro A. Normal mouse and rat strains as models for age-related cataract and the effect of caloric restriction on its development. Exp Eye Res. 2000;70(5):683–92. doi: 10.1006/exer.2000.0835 . 10870527

[33] Williams DS, Detre JA, Leigh JS, Koretsky AP. Magnetic resonance imaging of perfusion using spin inversion of arterial water. Proc Natl Acad Sci USA. 1992;89:212–6. doi: 10.1073/pnas.89.1.212 1729691 PMC48206

[34] Wong EC. An introduction to ASL labeling techniques. J Magn Reson Imaging. 2014;40(1):1–10. doi: 10.1002/jmri.24565 . 24424918

[35] Muir ER. Preclinical Arterial Spin Labeling Measurement of Cerebral Blood Flow. Methods Mol Biol. 2018;1718:59–70. doi: 10.1007/978-1-4939-7531-0_4 . 29341002

[36] Muir ER, Duong TQ. Layer-specific functional and anatomical MRI of the retina with passband balanced SSFP. Magn Reson Med. 2011;66(5):1416–21. doi: 10.1002/mrm.22935 . 21604296 PMC3161139

[37] Muir ER, Shen Q, Duong TQ. Cerebral blood flow MRI in mice using the cardiac spin-labeling technique. Magn Reson Med. 2008;60:744–8. doi: 10.1002/mrm.21721 18727091 PMC2581653

[38] Poulin MJ, Liang PJ, Robbins PA. Dynamics of the cerebral blood flow response to step changes in end-tidal PCO2 and PO2 in humans. J Appl Physiol (1985). 1996;81(3):1084–95. doi: 10.1152/jappl.1996.81.3.1084 . 8889738

[39] Regan RE, Duffin J, Fisher JA. Instability of the middle cerebral artery blood flow in response to CO2. PLoS One. 2013;8(7):e70751. doi: 10.1371/journal.pone.0070751 . 23936248 PMC3728315

[40] Lei B, Yao G, Zhang K, Hofeldt KJ, Chang B. Study of rod- and cone-driven oscillatory potentials in mice. Invest Ophthalmol Vis Sci. 2006;47(6):2732–8. doi: 10.1167/iovs.05-1461 . 16723493

[41] Li G, De La Garza B, Shih YY, Muir ER, Duong TQ. Layer-specific blood-flow MRI of retinitis pigmentosa in RCS rats. Exp Eye Res. 2012;101:90–6. doi: 10.1016/j.exer.2012.06.006 . 22721720 PMC3569723

[42] Chandra S, Muir ER, Deo K, Kiel JW, Duong TQ. Effects of Dorzolamide on Retinal and Choroidal Blood Flow in the DBA/2J Mouse Model of Glaucoma. Invest Ophthalmol Vis Sci. 2016;57(3):826–31. doi: 10.1167/iovs.15-18291 . 26934140 PMC4777278

[43] Armstrong RA. When to use the Bonferroni correction. Ophthalmic Physiol Opt. 2014;34(5):502–8. doi: 10.1111/opo.12131 . 24697967

[44] Harris A, Arend O, Danis RP, Evans D, Wolf S, Martin BJ. Hyperoxia improves contrast sensitivity in early diabetic retinopathy. Br J Ophthalmol. 1996;80(3):209–13. doi: 10.1136/bjo.80.3.209 8703857 PMC505430

[45] Drasdo N, Chiti Z, Owens DR, North RV. Effect of darkness on inner retinal hypoxia in diabetes. Lancet. 2002;359(9325):2251–3. doi: 10.1016/s0140-6736(02)09265-6 . 12103292

[46] Block F, Schwarz M. The b-wave of the electroretinogram as an index of retinal ischemia. Gen Pharmacol. 1998;30(3):281–7. doi: 10.1016/s0306-3623(97)00359-5 . 9510075

[47] Tummala SR, Benac S, Tran H, Vankawala A, Zayas-Santiago A, Appel A, et al. Effects of inhibition of neuronal nitric oxide synthase on basal retinal blood flow regulation. Exp Eye Res. 2009;89(5):801–9. doi: 10.1016/j.exer.2009.07.014 . 19646435

[48] Kaldi I, Dittmar M, Pierce P, Anderson RE. L-NAME protects against acute light damage in albino rats, but not against retinal degeneration in P23H and S334ter transgenic rats. Exp Eye Res. 2003;76(4):453–61. doi: 10.1016/s0014-4835(02)00334-2 . 12634110

[49] Granstam E, Granstam SO. Regulation of uveal and retinal blood flow in STZ-diabetic and non-diabetic rats; involvement of nitric oxide. Curr Eye Res. 1999;19(4):330–7. doi: 10.1076/ceyr.19.4.330.5300 . 10520229

[50] Li Q, Zemel E, Miller B, Perlman I. NADPH diaphorase activity in the rat retina during the early stages of experimental diabetes. Graefes Arch Clin Exp Ophthalmol. 2003;241(9):747–56. doi: 10.1007/s00417-003-0740-7 . 14564531

[51] Dmitriev AV, Henderson D, Linsenmeier RA. Development of diabetes-induced acidosis in the rat retina. Exp Eye Res. 2016;149:16–25. doi: 10.1016/j.exer.2016.05.028 . 27262608 PMC4969123

[52] Nowik M, Kampik NB, Mihailova M, Eladari D, Wagner CA. Induction of metabolic acidosis with ammonium chloride (NH4Cl) in mice and rats—species differences and technical considerations. Cell Physiol Biochem. 2010;26(6):1059–72. doi: 10.1159/000323984 . 21220937

[53] Wright WS, Yadav AS, McElhatten RM, Harris NR. Retinal blood flow abnormalities following six months of hyperglycemia in the Ins2(Akita) mouse. Exp Eye Res. 2012;98:9–15. doi: 10.1016/j.exer.2012.03.003 . 22440813 PMC3340465

[54] Blair NP, Wanek J, Felder AE, Brewer KC, Joslin CE, Shahidi M. Inner Retinal Oxygen Delivery, Metabolism, and Extraction Fraction in Ins2Akita Diabetic Mice. Invest Ophthalmol Vis Sci. 2016;57(14):5903–9. doi: 10.1167/iovs.16-20082 . 27802520 PMC5096417

[55] Peng Q, Zhang Y, Nateras OS, van Osch MJ, Duong TQ. MRI of blood flow of the human retina. Magn Reson Med. 2011;65(6):1768–75. doi: 10.1002/mrm.22763 . 21590806 PMC3197718

[56] San Emeterio Nateras O, Harrison JM, Muir ER, Zhang Y, Peng Q, Chalfin S, et al. Choroidal blood flow decreases with age: an MRI study. Curr Eye Res. 2014;39(10):1059–67. doi: 10.3109/02713683.2014.892997 . 24655028 PMC4241237

[57] Maccotta L, Detre JA, Alsop DC. The efficiency of adiabatic inversion for perfusion imaging by arterial spin labeling. NMR Biomed. 1997;10(4–5):216–21. doi: 10.1002/(sici)1099-1492(199706/08)10:4/5&lt;216::aid-nbm468&gt;3.0.co;2-u . 9430351

[58] Marro KI, Hayes CE, Kushmerick MJ. A model of the inversion process in an arterial inversion experiment. NMR Biomed. 1997;10(7):324–32. doi: 10.1002/(sici)1099-1492(199710)10:7&lt;324::aid-nbm491&gt;3.0.co;2-l . 9471123

[59] Sicard K, Shen Q, Brevard ME, Sullivan R, Ferris CF, King JA, et al. Regional cerebral blood flow and BOLD responses in conscious and anesthetized rats under basal and hypercapnic conditions: implications for functional MRI studies. J Cereb Blood Flow Metab. 2003;23(4):472–81. doi: 10.1097/01.WCB.0000054755.93668.20 . 12679724 PMC2989608

[60] Brian JE, Bogan L, Kennedy RH, Seifen E. The impact of streptozotocin-induced diabetes on the minimum alveolar anesthetic concentration (MAC) of inhaled anesthetics in the rat. Anesth Analges. 1993;77:342–5. doi: 10.1213/00000539-199377020-00022 8346836

[61] Munting LP, Derieppe M, Suidgeest E, Hirschler L, van Osch MJ, Denis de Senneville B, et al. Cerebral blood flow and cerebrovascular reactivity are preserved in a mouse model of cerebral microvascular amyloidosis. Elife. 2021;10:e61279. doi: 10.7554/eLife.61279 . 33577447 PMC7880694

[62] Venkataraman ST, Hudson C, Fisher JA, Rodrigues L, Mardimae A, Flanagan JG. Retinal arteriolar and capillary vascular reactivity in response to isoxic hypercapnia. Exp Eye Res. 2008;87(6):535–42. doi: 10.1016/j.exer.2008.08.020 . 18840429

[63] Campen MJ, Tagaito Y, Li J, Balbir A, Tankersley CG, Smith P, et al. Phenotypic variation in cardiovascular responses to acute hypoxic and hypercapnic exposure in mice. Physiol Genomics. 2004;20(1):15–20. doi: 10.1152/physiolgenomics.00197.2003 . 15494473

[64] Campen MJ, Tagaito Y, Jenkins TP, Balbir A, O’Donnell CP. Heart rate variability responses to hypoxic and hypercapnic exposures in different mouse strains. J Appl Physiol (1985). 2005;99(3):807–13. doi: 10.1152/japplphysiol.00039.2005 . 15890760

[65] Gurley SB, Mach CL, Stegbauer J, Yang J, Snow KP, Hu A, et al. Influence of genetic background on albuminuria and kidney injury in Ins2(+/C96Y) (Akita) mice. Am J Physiol Renal Physiol. 2010;298(3):F788–95. doi: 10.1152/ajprenal.90515.2008 . 20042456 PMC2838602

[66] Basu R, Oudit GY, Wang X, Zhang L, Ussher JR, Lopaschuk GD, et al. Type 1 diabetic cardiomyopathy in the Akita (Ins2WT/C96Y) mouse model is characterized by lipotoxicity and diastolic dysfunction with preserved systolic function. Am J Physiol Heart Circ Physiol. 2009;297(6):H2096–108. doi: 10.1152/ajpheart.00452.2009 . 19801494

[67] Constantinides C, Mean R, Janssen BJ. Effects of isoflurane anesthesia on the cardiovascular function of the C57BL/6 mouse. ILAR J. 2011;52(3):e21–31. . 21677360 PMC3508701

